# The Injection of Pulmonary Cavities

**Published:** 1875-01

**Authors:** 


### THE INJECTION OF PULMONARY CAVITIES.

Professor Pepper, of Philadelphia, who contributed a paper to the Philadelphia *Medical Times* in March last, on the local treatment of pulmonary cavities by injections through the chest-wall, has given the results of further experience of this method of treatment in an article in the October number of the *American Journal of the Medical Sciences.* We pass over historical references as to the early suggestions of, and priority in, the resort to this method of treatment, to state in abstract, that he uses a very delicate steel canulated needle, like the finest hypodermic needles, but about three inches in length, and an hypodermic syringe capable of holding twenty-five minims. He at first used an aspirator, but now prefers the instrument mentioned. Selecting a point at which the signs of a cavity are most intense, he punctures the chest-wall, previously affecting anæsthesia, by freezing. There is little or no pain except when the filaments of a nerve are pricked, when tingling radiating pains are felt. The time occupied in an injection does not exceed thirty seconds. The depth to which it has been necessary to penetrate has varied in different cases, from one-and-a-half to two inches. Prof. Pepper has only used dilute solutions of iodine in iodide of potassium (Lugol's Solution), and says that the results of injections of iodine have been so satisfactory that he has felt indisposed to use any other substance, though he considers it probable that other substances may be found preferable in some cases. Twelve minims of the liquor iodini comp. are diluted in a drachm of warm water, and of this solution twenty-five minims are injected about once a week. He reports six cases treated in the Philadelphia Hospital, three of which were not benefited, the other three being considerably improved. He claims that the continuous treatment of lung cavities by repeated injections by means of delicate canulæ may be conducted without hemorrhage, traumatic irritation, or interference with internal medication and hygienic measures. The cases which are best adapted for this local treatment are those where a single, superficial and circumscribed non-tuberculous cavity exists ; but even where there is implication of the rest of the lung, or incipient disease of the opposite lung, some benefit may be expected. The mode in which such local treatment does good is chiefly by altering the character of the morbid action in the walls of the cavity, diminishing the amount of purulent formation, as well as the degree of hectic irritation and the danger of constitutional infection. A certain amount of rest is secured for the walls of the cavity by the marked relief afforded to the cough. Further, the treatment favors the cicatrization and contraction of cavities. He finally maintains that this mode of treatment possesses a certain degree of positive clinical value, since, during its use, uniform improvement to an exceptional extent has taken place in both the general and local conditions of the patient.

In a matter of such immense importance as this question of the curability of lung cavities, the interest of Prof. Pepper's paper will not be underrated. Without being over-sanguine concerning the results as yet incomplete though promising, the experimental tests recorded warrant a further and extended trial, which the practice will undoubtedly receive.—*Canada Lancet.*

